# Gait recognition using spatio-temporal representation fusion learning network with IMU-based skeleton graph and body partition strategy

**DOI:** 10.1371/journal.pone.0332947

**Published:** 2025-10-08

**Authors:** Fo Hu, Qinxu Zheng, Xuanjie Ye, Zukang Qiao, Junlong Xiong, Hongsheng Chang

**Affiliations:** 1 Institute of Wenzhou, Zhejiang University, Wenzhou, People’s Republic of China; 2 Zhejiang University, Hangzhou, People’s Republic of China; 3 Zhejiang University of Technology, Hangzhou, People’s Republic of China; 4 The First Affiliated Hospital of Zhejiang Chinese Medical University (Zhejiang Provincial Hospital of Chinese Medicine), Hangzhou, People’s Republic of China; Polytechnic University of Marche: Universita Politecnica delle Marche, ITALY

## Abstract

The precise recognition of human lower limb movements based on wearable sensors is very important for human-computer interaction. However, the existing methods tend to ignore the dynamic spatial information in the process of executing human lower limb movements, leading to challenges such as reduced decoding accuracy and limited robustness. In this paper, we construct skeleton graph data based on inertial measurement unit (IMU) sensors. Also, a two-branch deep learning model, termed TCNN-MGCHN, is proposed to mine meaningful spatial and temporal feature representations from IMU-based skeleton graph data. Firstly, a temporal convolutional module (consisting of a multi-scale convolutional sub-module and an attention sub-module) is developed to extract temporal feature information with highly discriminative power. Secondly, a multi-scale graph convolutional module and a spatial graph edges’ importance weight assignment method based on body partitioning strategy are proposed to obtain intrinsic spatial feature information between different skeleton nodes. Finally, the fused spatio-temporal features are passed into the classification module to obtain the predicted gait movements and sub-phases. Extensive comparison and ablation studies are conducted on our self-constructed human lower limb movement dataset. The results demonstrate that TCNN-MGCHN delivers superior classification performance compared to the mainstream methods. This study can provide a benchmark for IMU-based human lower limb movement recognition and related deep-learning modeling works.

## Introduction

With the advancements in computer performance and data processing algorithms, human-computer interaction has progressed into a rapid development stage [1–5]. As an essential part of human-computer interaction technology, gait recognition method is widely used in medical rehabilitation [6–8], identity recognition [9], virtual reality [10], and other fields. At present, gait can be identified from various data forms (physiological signals [11–13], video streams [14,15], skeleton graph data [16,17], etc.). In particular, skeleton graph data has obvious advantages over other data types: even in complex environments, skeleton graph data retains sufficient kinematic and spatial information with good robustness. Therefore, research on gait recognition based on skeleton graph data has received much attention. However, skeleton graph data usually requires pre-installed vision equipment and thus cannot be used outside of the equipment’s working area. The inertial measurement unit (IMU) can overcome the limitation of the fixed area well and can provide abundant motion information. However, a single IMU sensor cannot provide abundant spatial information. Therefore, it is crucial to efficiently lay out multiple IMU sensors and establish a portable skeleton graph construction method.

In addition, IMU signals are non-stationary, weak, and low-frequency, resulting in insufficient decoding ability of traditional machine learning methods (naive Bayes [18], hidden Markov model [19], decision tree [20], support vector machine [21], etc.). With the rapid development of deep learning, many studies have applied it to the field of gait recognition. Deep learning has been proven to generally outperform traditional machine learning methods, significantly improving the accuracy of gait recognition [22]. Deep learning mainly includes two methods based on Euclidean data and non-Euclidean data. The deep learning methods based on Euclidean data include the convolutional neural network (CNN) [23], recurrent neural network (RNN) [24], and their variants. Zhao et al. [25] converted IMU data into angle embeddFed gait dynamic images, designed a shallow (3-layer) CNN model, and tested it on the two open gait movement databases. The results show that the average recognition accuracy of their model on the different day testing is only 67.9%, and the generalization ability is poor. Obviously, due to the inherent complexity of human movement, it is difficult for the shallow model to learn the nonlinear relationship from human motion data. Therefore, many studies have started to explore deep neural network methods. Arshad et al. [26] converted IMU signals into granular angular field images, designed a deep CNN model, and implemented a sense-based frailty assessment with an average recognition accuracy of 85.1%. However, IMU data is a time series signal with obvious time dependence relationship. Neither shallow nor deep CNN models can effectively capture the time-dependent relationships in the time-series signal. Therefore, some researchers [27,28] have used RNN and its variant models, which are good at extracting temporal features, for gait recognition. However, RNN and its variant models are challenging to learn local spatial features in IMU signals, and their improvement in the accuracy of gait recognition is limited. Therefore, various pattern recognition methods combining CNN and RNN have been proposed successively [29,30]. Compared with a single network model, the CNN-RNN model effectively combines the advantages of the two models and has been widely concerned. Although the CNN-RNN model has achieved some improvement in the accuracy of gait recognition, its ability to extract spatial feature information is limited. Constructing an effective deep learning model to improve the feature mining ability of IMU-based skeleton graph data is still a critical problem to be urgently solved. However, the skeleton graph is a kind of non-Euclidean data with highly correlated feature information among skeleton nodes. Classical CNN, RNN, and even CNN-RNN methods cannot extract the spatial feature information among skeleton nodes. Therefore, deep learning methods based on non-Euclidean data have received extensive attention. Graph Convolutional Network (GCN) [31,32] can perform convolution operation on the graph data, which has excellent recognition performance for classification tasks based on skeleton graph data. Yan et al. [33] first proposed the spatial-temporal convolutional network to model skeleton graph data from spatial-temporal domains. Also, some studies have showed that GCN has strong spatial information modeling capacity [33,34], but its temporal information mining ability still needs to be improved. Generally speaking, existing deep learning methods can only extract a portion of the feature information of human motion data with limited expressive power and generalization difficulties. Therefore, it is crucial to establish a deep learning-based skeleton graph data decoding method with more remarkable expression and generalization ability for gait and sub-phase recognition.

As highlighted above, the field of human lower limb movement recognition using multiple IMU sensors has several critical shortcomings. Firstly, traditional machine learning methods often struggle with decoding non-stationary, weak, and low-frequency IMU signals effectively, limiting their applicability in dynamic and complex movement scenarios. Secondly, while both shallow and deep convolutional neural networks (CNNs) have been explored, they exhibit significant limitations in capturing the essential time-dependent relationships inherent in IMU signals, resulting in suboptimal performance for sequential data analysis. Thirdly, recurrent neural networks (RNNs) and their variants, although adept at handling temporal dependencies, face substantial challenges in learning and representing local spatial features within the IMU data, which is crucial for accurate gait analysis. Finally, there is a pressing need for advanced methods capable of extracting and utilizing spatial feature information among skeleton nodes in non-Euclidean data structures, as conventional approaches often fall short in modeling the complex interrelationships present in such data. Addressing these issues is vital for advancing the accuracy of human movement recognition systems.

To address the issues above, we propose a multi-branch deep learning network called the temporal convolutional neural network and multi-scale dynamic graph convolutional hybrid network (TCNN-MGCHN) for accurate gait and sub-phases recognition based on skeleton graph data. Our model leverages a temporal convolutional module (TCM) to capture highly discriminative temporal features from IMU signals. Also, we introduced a multi-scale graph convolutional module (MGCM) combined with a body partitioning strategy to enhance the extraction of intrinsic spatial feature information between different skeleton nodes. In addition, the fused spatio-temporal features from our model improve classification performance for gait movements and sub-phases, demonstrating superior accuracy compared to mainstream methods. The main contributions of this paper are summarized as follows:

1. We design a multi-task gait experiment and propose portable skeleton graph data construction method based on multiple IMU sensors.
2. We propose a spatial partitioning strategy to effectively explore the inherent spatial information among multiple IMU signals, thereby enhancing the spatial feature extraction capabilities of GCN.
3. TCNN-MGCHN demonstrates strong human lower limb movement recognition performance and effective spatio-temporal feature fusion extraction. Experimental results validate that TCNN-MGCHN outperforms state-of-the-art methods on the self-made datasets.

## Materials and methods

### Experimental design

Participants were recruited between June and December 2023 through an online forum, and the experimental protocol was designed in accordance with the regulations of the local ethics review committee. To ensure the validity and consistency of the gait recognition experiments, all participants were required to meet the following inclusion criteria: (1) No history of musculoskeletal disorders affecting the lower limbs (e.g., fractures, joint instability, or chronic pain); (2) no known neurological or motor function disorders that could affect fine motor control or movement coordination of the lower limbs. Ultimately, a total of 23 participants were recruited to participate in the multi-task gait experiment, including 13 males (height: 172±1.24 cm, age: 26.32±2.19 years) and 12 females (height: 161±1.47 cm, age: 23.72±1.54 years). Before the experiment, they were also asked to eat proper food and get enough sleep. In addition, subjects will receive cash compensation after completing the experiment. Each participant read and signed a written informed consent form approved by the Ethics Committee of Zhejiang University of Technology under Application No. ZJUT-2023-47.

**Fig 1:**
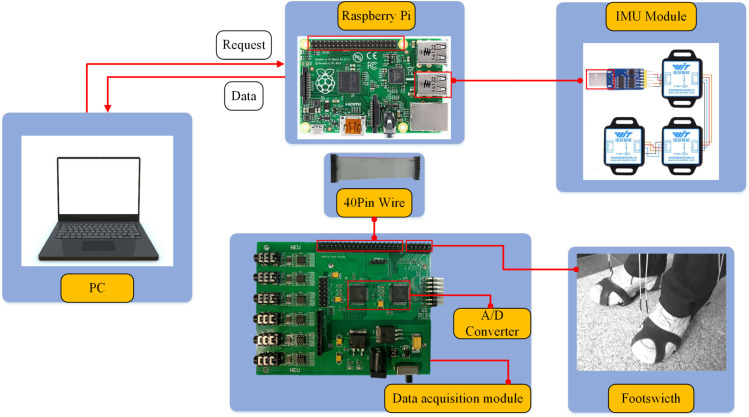
Multi-modal signals acquisition device.

**Fig 2:**
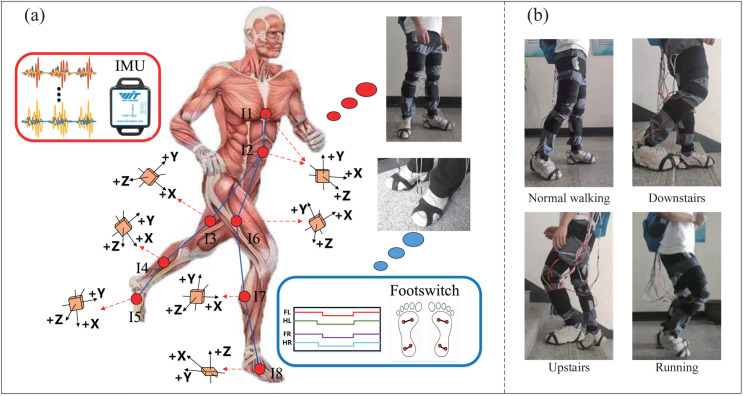
Multi-task gait experiment and sensor layout.

A multi-modal signal acquisition device was employed to record IMU and foot switch signals. The foot switch signals and IMU data were synchronously acquired using the same multi-modal signal acquisition device. As illustrated in Fig 1, the device comprises three modules: a laptop, a Raspberry Pi, and a data acquisition module. Specifically, we placed eight IMU sensors (WT901C485, Shenzhen Vit Intelligent Technology Co., LTD.) with a sampling frequency of 100 Hz on the human body: chest, thigh, calf, and ankle. The accelerometer range was ±16g, and the gyroscope range was ±2000°/s. Fig 2 shows the placement and triaxial orientation of the eight IMU sensors (I1-I8). It is worth noting that, to better capture the upper-body movements, two IMU sensors were installed on the chest. This dual-sensor setup enables more accurate monitoring of subtle trunk motions, including rotations and asymmetrical movements, thereby providing higher spatial resolution for subsequent motion data analysis. Moreover, in the anatomical standing posture, the local reference frames of all IMUs (x, y, and z axes) were oriented identically to that of sensor I1. The orientation definitions in the body-centered coordinate system were as follows: 1) The x-axis was defined as the horizontal axis aligned with the shoulders, pointing from the center of the torso toward the left side of the body, 2) the y-axis was the vertical axis aligned with the spine, pointing upward toward the head, 3) the z-axis was perpendicular to the chest, pointing anteriorly, i.e., forward in the direction the body is facing. Consistent with the analysis scheme of study [35], we use the triaxial angular rate signal as the analytical physical quantity. In addition, we used a wearable foot switch shoe cover made of thermoplastic polyurethane material. The shoe cover fits tightly to the sole, which can make the sensor highly sensitive in use, as shown in Fig 2. It should be noted that we place two parallel connected foot switch sensors at the front foot and heel respectively, which can expand the response area of the sensors. In total, we collected four-channel foot switch signals with a sampling frequency of 1000 Hz, including two channels for each of the left and right foot.

**Fig 3:**
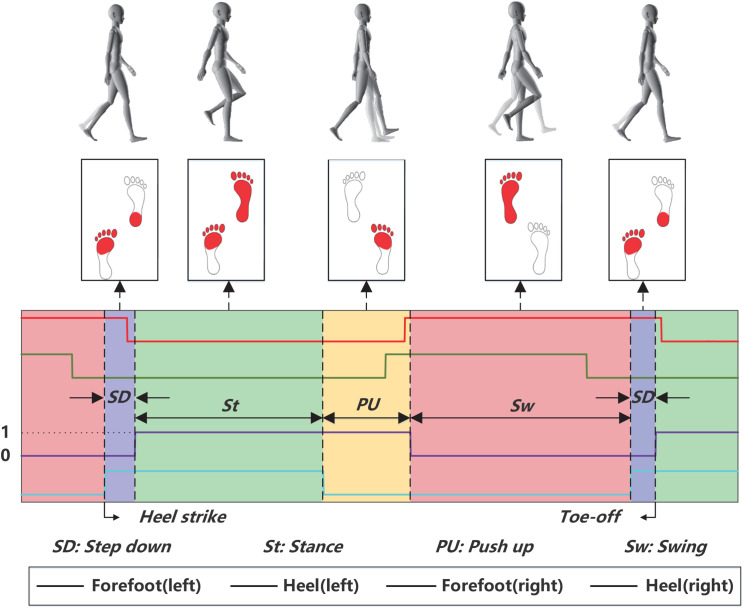
Sub-phase division method for gait.

The experiment was conducted on an open flat area on the Zhejiang University of Technology campus. As shown in Fig 2, subjects were required to complete four gait tasks: Normal walking, downstairs, upstairs, and running. Fig 3 illustrates the sub-phase division method for gait. Here, Forefoot ‘(right)’ and ‘Heel (right)’ represent the footswitch recordings for the right forefoot and right heel, respectively. The gait cycle is subsequently divided into four stages based on the footswitch recordings: step down (SD), stance (St), push-up (PU), and swing (Sw). All subjects performed the gait at their normal pace, with each session lasting ten minutes.

### Data preprocessing

The raw angular velocity signal contains a lot of artifacts and noise, so it is necessary to design an effective signal preprocessing algorithm to improve the signal quality. Specifically, a second-order Butterworth filter with a cutoff frequency of 10 Hz was applied to the angular velocity signal to eliminate high-frequency noise. Then, to preserve the high temporal resolution of the foot switch events, we resampled the angular rate signals to 1000 Hz. This is crucial for precise gait phase detection while maintaining consistent sampling rates across different modalities for accurate data fusion. Each foot switch sensor has two states: “ON” or “OFF”, where “ON” means that the foot switch sensor is in contact with the ground, and “OFF” is the opposite, as shown in Fig 3.

The foot switch is the most commonly used analytical signal for human movement sub-phase division. Additionally, in this study, the Z-axis of the IMU sensor positioned on the human lower limb is orthogonal to the sagittal plane of the body, providing sufficient kinematic information through the angle signal in this direction. Therefore, we use the Z-axis angle signal $A_{af}^{Z}$ of the IMU sensor located at the thigh of the active foot (the power foot on takeoff) and the foot switch signals of both feet to realize the definition and division of the gait sub-phases, as shown in Fig 3. This study includes one gait classification task and four gait sub-phase classification tasks. Both the gait classification task and each of the gait sub-phase classification tasks comprise four distinct categories.

Next, we use a sliding window to intercept sample data segments from angular rate signals with 8×3 channels. The sliding window utilized in this study has a length of 200 data points and a step size of 40 data points. Therefore, the input size is 8×3×200. Since the delay caused by data interception is less than 300 ms, the data interception scheme is considered to be sufficient to ensure that the model can perform classification tasks continuously and in real time [30]. In addition, sample balancing operations are performed to ensure that the model is evaluated accurately.

### Methods

In this section, the proposed TCNN-MGCHN framework and algorithm are introduced in **Model architecture**. Subsequently, We provide the implementation details of the TCNN-MGCHN model.

#### Model architecture.

**Fig 4:**
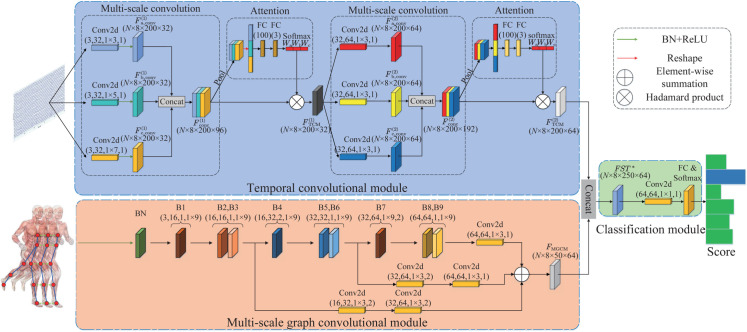
Overall architecture of the TCNN-MGCHN model, which consists of three modules: TCM, MGCM, and classification module. The kernel parameter sizes used in each module are explicitly annotated within the figure.

As illustrated in Fig 4, an end-to-end TCNN-MGCHN model was designed for human lower limb movement (HLLM) and sub-phase recognition, utilizing the spatio-temporal feature distribution of IMU-based skeleton graph data. The TCNN-MGCHN model consists of three functional modules: A TCM, a MGCM, and a classification module (CM). The TCM captures temporal dependencies in IMU signals by applying convolutional operations along the temporal dimension, enabling the model to learn high-level features essential for accurate movement recognition. The MGCM captures spatial relationships among skeleton nodes in a non-Euclidean space using multi-scale graph convolutional operations to learn both local and global features. The body partitioning strategy enhances this process by dividing the skeleton into regions, allowing the MGCM to focus on specific areas and capture detailed spatial information, leading to a more holistic understanding of movement patterns. The Classification Module fuses temporal and spatial features from the TCM and MGCM, creating a comprehensive spatio-temporal representation that enhances accuracy and robustness in gait recognition. In the following, we detail the specific model architecture.

**TCM:** The TCM consists of a multi-scale convolutional sub-module and an attention sub-module. We transform angular velocity signal to learn high-level temporal domain features. To ensure that the TCM can accurately capture strongly discriminative temporal domain features, we adopt convolution layers with different convolution kernel sizes to automatically extract salient pattern at multiple time scales. Then, the attention sub-module is then used to adaptively learn and focus on important time-scale feature information. As shown in Fig 4, two TCM are used in this paper, and the output feature map of the *j*th TCM can be represented as $F_{TCM}^{\left(j\right)}\in R^{N\;\times \;S\;\times \;T\;\times \;w_{j}}$. The parameters of the TCM are shown in Fig 4, and we take the convolutional module whose parameters are (3,32,1×3,1) as an example to illustrate: In this context, 3 denotes the input channel dimension, 32 represents the output channel dimension of the convolutional layer, 1×3 specifies the convolution kernel size, and 1 indicates the stride of the convolution. The TCM receives pre-processed IMU signals, including accelerometer and gyroscope data, segmented into fixed-length windows, forming multi-dimensional tensors $\hat{X}\in R^{S\;\times \;T\;\times \;C}$ with dimensions representing time steps *T*, IMU features *C*, and sensor count *S*, capturing the temporal dynamics of gait.

Multi-scale convolutional sub-module: Assume that the input data is $X=\left[x_{1},\ldots ,x_{N}\right]\in R^{N\;\times \;S\;\times \;T\;\times \;C}$, $x_{N}\in R^{S\;\times \;T\;\times \;C}$, where *S* represents the number of skeleton nodes, *C* is the number of channels per skeleton node, and each channel has *T* data samples. In the *j*th TCM, we first perform three convolution operations on the input data *X*: $\xi_{1}:X\to F_{a0}^{\left(j\right)}\in R^{N\;\times \;S\;\times \;T\;\times \;w_{j}}$, $\xi_{2}:X\to F_{b0}^{\left(j\right)}\in R^{N\;\times \;S\;\times \;T\;\times \;w_{j}}$, and $\xi_{3}:X\to F_{c0}^{\left(j\right)}\in R^{N\;\times \;S\;\times \;T\;\times \;w_{j}}$, respectively, to obtain the low-scale, medium-scale, and high-scale temporal features $F_{a0}^{\left(j\right)}$, $F_{b0}^{\left(j\right)}$, and $F_{c0}^{\left(j\right)}$. Then, features $F_{a0}^{\left(j\right)}$, $F_{b0}^{\left(j\right)}$, and $F_{c0}^{\left(j\right)}$ are successively processed through batch normalization (BN) and ReLU layer to obtain features $F_{a1}^{\left(j\right)}\in R^{N\;\times \;S\;\times \;T\;\times \;w_{j}}$, $F_{b1}^{\left(j\right)}\in R^{N\;\times \;S\;\times \;T\;\times \;w_{j}}$, and $F_{c1}^{\left(j\right)}\in R^{N\;\times \;S\;\times \;T\;\times \;w_{j}}$ to accelerate the model learning process and alleviate the vanishing gradient problem. Finally, we concatenate the features $F_{a1}^{\left(j\right)}$, $F_{b1}^{\left(j\right)}$, and $F_{c1}^{\left(j\right)}$ to generate features $F_{conc}^{\left(j\right)}\in R^{N\;\times \;S\;\times \;T\;\times \;3w_{j}}$.

Attention sub-module: In this sub-module, we fuse multi-scale temporal features and use the attention layer to pay attention to important skeleton nodes and channels. First, we perform pooling operations on features $F_{conc}^{\left(j\right)}$ to generate features $F_{conc,p}^{\left(j\right)}$ to reduce the feature dimension and avoid overfitting. Then, the features $F_{p}^{\left(j\right)}$ are flattened to one-dimensional vectors $F_{f}^{\left(j\right)}$. Next, feature $F_{f}^{\left(j\right)}$ is passed through two fully connected (FC) layers followed by a softmax layer, yielding the attention weights $w_{a}^{\left(j\right)}$, $w_{b}^{\left(j\right)}$ and $w_{c}^{\left(j\right)}$ of features $F_{conc}^{\left(j\right)}$ can be obtained. Finally, we multiply the obtained attention weights with the multi-time scale temporal features to obtain the final fusion temporal feature map $F_{TCM}^{\left(j\right)}$:

$$F_{TCM}^{\left(j\right)}=F_{conc}^{\left(j\right)}⊗\left(w_{a}^{\left(j\right)},w_{b}^{\left(j\right)},w_{c}^{\left(j\right)}\right),w_{a}^{\left(j\right)}+w_{b}^{\left(j\right)}+w_{c}^{\left(j\right)}=1,$$ (1)

where ⊗ denotes Hadamard product. In summary, the TCM can fuse features at multiple time scales and adaptively focus on important nodes and channels of features, making the temporal features extracted by the model have more classification performance and generalization ability.

**MGCM:** As mentioned above, we constructed the skeleton graph data based on eight IMU sensors. Then, the MGCM is designed to learn the spatio-temporal feature representations from skeleton graph data.

**Fig 5:**
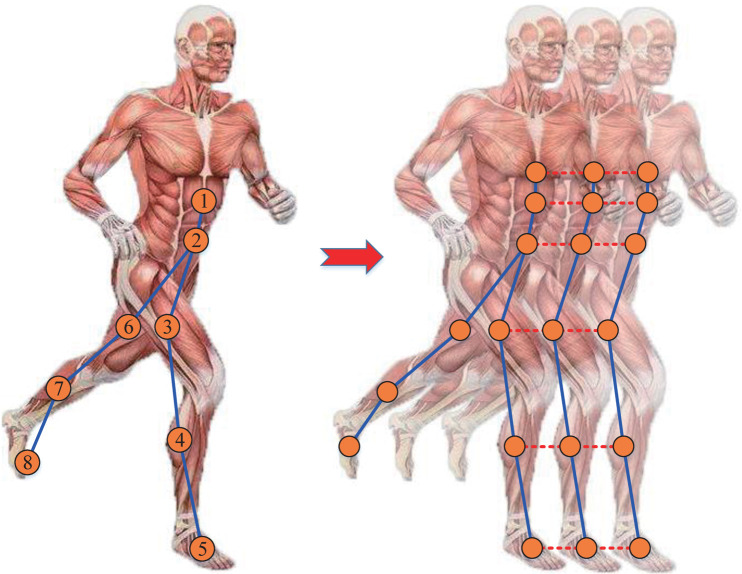
Illustration of the constructed skeleton graph data.

Skeleton graph data construction: Fig 5 shows the constructed skeleton graph, where the IMU sensors are used as skeleton nodes and the three-axis angular rate signals are used as feature vectors for each skeleton node. The sensor numbers are labeled with digits. The blue lines between the skeleton nodes represent the natural connection of the body joints. In addition, we connect the adjacent sample points of the same skeleton node to make the data retain the time information, as shown by the red dashed line in Fig 5.

Suppose that a single sample point’s spatio-temporal skeleton graph data is represented as G={V,E}, where *V* is the set of s(s=8) skeleton nodes and *E* is the set of edges. In this paper, each skeleton node has a *m*(*m* = 3) dimensional feature. Therefore, the skeleton node feature matrix of a single sample point can be represented as $X\in R^{s\;\times \;m}$, and the adjacency matrix can be represented as $A_{ij}\in {\left\{0,1\right\}}^{s\;\times \;s}$. Then, $A_{ij}$ can be defined as follows:

$$A_{ij}=\left\{ \begin{array}{c} \begin{array}{cc} 1 & if\;\;i=j\;\;or\;\;node\;\;i\;\;is\;\;connected\;\;to\;\;node\;\;j \end{array}\\ \begin{array}{cc} 0 & if\;\;node\;\;i\;\;is\;\;not\;\;connected\;\;to\;\;node\;\;j \end{array} \end{array}\right..$$ (2)

*A* is then utilized to compute the Laplacian matrix L, as shown in Eq (3).

$$L={\hat{D}}^{-1/2}\hat{A}{\hat{D}}^{-1/2},$$ (3)

where $\hat{A}=A+I_{N}$, $I_{N}$ is the identity matrix. $\hat{D}=diag\left(\left[d_{1},\ldots ,d_{i},d_{n}\right]\right)$ is degree matrix of the vertices. Eq (4) illustrates the GCN updating process for each layer.

$$O_{G}=ReLU\left(Lx_{N}\theta \right)\in R^{S^{\prime }\;\times \;T\;\times \;C\;\times \;G},$$ (4)

where $x_{N}\in R^{S\;\times \;T\;\times \;C}$ denotes the input data. $\theta \in R^{S^{\prime }\;\times \;T\;\times \;C\;\times \;G}$ represents the learnable parameter matrix of the GCN filter. *G* denotes the output dimension of the feature after the GCN layer.

**Fig 6:**
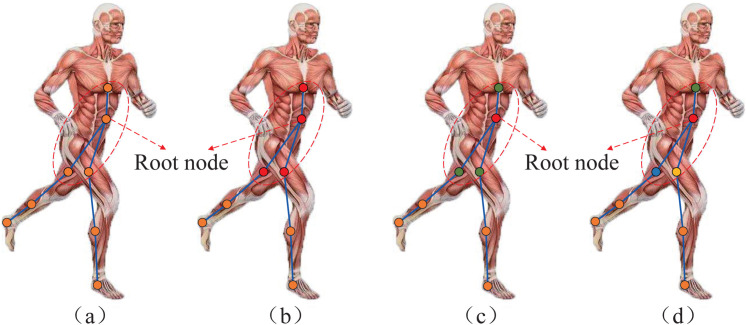
Three partitioning strategies. From left to right: (a) Single frame skeleton graph (b) Uni-label partition strategy. (c) Dual-label partition strategy. (d) Body partition strategy.

According to Eq (4), the parameter *θ* is shared in the graph convolution operation without considering the effect of different body regions on the edge weight parameters. Therefore, we design three partitioning strategies to enhance high-level spatio-temporal feature extraction capability of graph convolutional networks: Uni-label partitioning strategy, dual-label partitioning strategy, and body partitioning strategy. As shown in Fig 6(a), the root node and neighbor nodes constitute a neighbor set $B\left(v_{i}\right)$, marked in the red dotted box. Unlike the conventional graph convolution method, we set a partitioning strategy to divide the neighbor set $B\left(v_{i}\right)$ into *K* subsets. Then, we can divide the nodes in set $B\left(v_{i}\right)$ to the corresponding subset label by mapping $l_{i}:B\left(v_{i}\right)\to \left\{1,\ldots ,K\right\}$. Therefore, the weight parameter can be expressed by the following equation:

$$w\left(v_{i},v_{j}\right)=w\prime \left(l_{i}\left(v_{j}\right)\right).$$ (5)

Uni-label partition strategy: This strategy treats the neighbor set $B\left(v_{i}\right)$ as a whole. Employing this strategy is equivalent to computing the inner product between the weight vector and the average feature vector of the root node along with all adjacent nodes. As shown in Fig 6(b), all nodes of the neighbor set $B\left(v_{i}\right)$ have the same label (red). This strategy is sub-optimal because it may ignore other body regions’ differences and interaction information. In this case, *K* = 1 and $l_{i}\left(v_{j}\right)=1,\forall i,j\in V$.

Dual-label partitioning strategy: This strategy treats the neighbor set $B\left(v_{i}\right)$ as two parts: the root node and its neighbor nodes. Assume that the distance from node $v_{j}$ to the root node $v_{i}$ is $d_{ij}$, where $d_{ij}=0$ represents the root node itself, while the remaining neighboring nodes are located in the $d_{ij}=1$ subset. As shown in Fig 6(c), the root node is red, and its neighbor nodes are green. Therefore, the neighbor set $B\left(v_{i}\right)$ will have two different weight vectors which can model the local electrode interaction information. Formally, we have *K* = 2 and $l_{i}\left(v_{j}\right)=d_{ij}$.

Body partitioning strategy: According to the spatial distribution characteristics and functional properties of brain electrodes, $B\left(v_{i}\right)$ was divided into three subsets: 1) $v_{i}$ itself, 2) trunk region (TR): The nodes $v_{j}$ connected to root node in the trunk region, 3) left lower limb region (LLLR): The nodes $v_{j}$ connected to root node in the left lower limb region. 3) right lower limb region (RLLR): The nodes $v_{j}$ is connected to the root node in the right lower limb region. This strategy is inspired by differences in the functions of various body regions. The trunk stabilizes the center of gravity, generates and transmits force. The left and right lower limbs support and move the body, and there are subtle differences in their function. As shown in Fig 6(d), the root node $v_{i}$ is red, the nodes $v_{j}$ connected to $v_{i}$ in the TR are green, the nodes $v_{j}$ connected to $v_{i}$ in the LLLR are blue, and the nodes $v_{j}$ connected to $v_{i}$ in the RLLR are yellow. Formally, we have *K* = 4 and $l_{i}\left(v_{j}\right)$ is calculated as follows:

$$l_{i}\left(v_{j}\right)=\left\{ \begin{array}{c} \begin{array}{cc} 1 & if\;\; \end{array}v_{j}\;\;is\;\;root\;\;node\\ \begin{array}{cc} 2 & if\;\; \end{array}v_{j}\;\;is\;\;connected\;\;to\;\;v_{i}\;\;and\;\;v_{j}\in TR\\ \begin{array}{cc} 3 & if\;\; \end{array}v_{j}\;\;is\;\;connected\;\;to\;\;v_{i}\;\;and\;\;v_{j}\in LLLR\\ \begin{array}{cc} 4 & if\;\; \end{array}v_{j}\;\;is\;\;connected\;\;to\;\;v_{i}\;\;and\;\;v_{j}\in RLLR \end{array}\right.$$ (6)

Fig 6 provides a visualization of the three partitioning strategies. We will evaluate the effectiveness of the proposed body partitioning strategy in Sect 4. More advanced partitioning strategies are expected to lead to better modeling capabilities and recognition performance. By combining the graph construction method and the partition strategy, each layer’s GCN update process can be expressed as follows:

$$S=\mathrm{ReLU}\left(LXw^{\prime }l_{i}\left(v_{j}\right)\right)\in R^{S^{\prime }\;\times \;T\;\times \;C\;\times \;G},$$ (7)

where $x_{N}\in R^{S\;\times \;T\;\times \;C}$ is the input data and *G* denotes the output dimension of the feature after the GCN layer.

**Fig 7:**
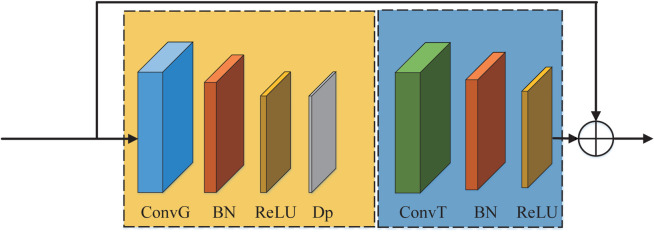
Spatial-temporal graph convolutional block’s architecture. ConvG denotes graph convolutional layer and ConvT denotes temporal convolutional layer.

As shown in Fig 4, the backbone structure of the multi-branch GCN designed in this paper consists of BN layers and nine graph convolutional blocks (B1, B2, ..., B9). The spatio-temporal graph convolutional block comprises a graph convolutional layer, followed by a batch normalization (BN) layer, a ReLU activation function, a dropout (Dp) layer, and a temporal convolutional layer, with an additional BN layer and ReLU function, as shown in Fig 7. As shown in Fig 4, the parameters of block B1 are (3,16,1, 1×9), where 3 represents the input channel dimension, 16 denotes the output channel dimension of spatio-temporal graph convolutional block, 1 is the convolution kernel size for the graph convolutional layer, and 1×9 specifies the convolution kernel size for the temporal convolution layer. In B1, B2, B3, B5, B6, B8, and B9, the stride of the temporal convolutional layers is set to 1. In blocks B4 and B7, the stride of the temporal convolutional layer is set to 2 to reduce the number of parameters of the features. Initially, BN is applied to normalize the input data $X=\left[x_{1},\ldots ,x_{n}\right]\in R^{N\;\times \;S\;\times \;T\;\times \;C}$, $x_{n}\in R^{S\;\times \;T\;\times \;C}$, yielding feature $F_{g0}\in R^{N\;\times \;S\;\times \;T\;\times \;C}$. Then, the feature $F_{g0}$ is then fed into multiple spatio-temporal graph convolutional blocks to extract the spatial feature representations. After nine spatio-temporal graph convolution operations and a convolution operation in the backbone structure, we can obtain the high-order feature $F_{m9}\in R^{n\;\times \;S\;\times \;T/4\;\times \;W}$, *W* = 64. In addition, we design a branch from B3 and B6 blocks respectively to obtain intermediate features $F_{b3}$ and $F_{b6}$, and then pass $F_{b3}$ and $F_{b6}$ through two convolutional layers to obtain features $F_{b3}^{\prime }$ and $F_{b6}^{\prime }$, respectively. The convolutional layer parameters of the backbone and the two branches are shown in Fig 4. Finally, we can obtain the final fusion output $F_{MGCM}\in R^{n\;\times \;S\;\times \;T/4\;\times \;W}$ of MGCM by element-wise summation calculation of features $F_{m9}$, $F_{b3}^{\prime }$ and $F_{b6}^{\prime }$:

$$F_{DGC}=\left\{ \begin{array}{c} F_{m9}⊕F_{b3}^{\prime }⊕F_{b6}^{\prime }\\ F_{b3}^{\prime }=Conv\left(Conv\left(F_{b3}\right)\right)\\ F_{b6}^{\prime }=Conv\left(Conv\left(F_{b6}\right)\right) \end{array}\right..$$ (8)

where Conv(·) is the convolution function and ⊕ denotes element-wise summation operation. Therefore, we obtain the spatial feature $F_{MGCM}$ from MGCM. Additionally, the adjacency matrix *A* of MGCM is continuously updated throughout the network training process. Algorithm 1 introduces the process of dynamical updating *A*.

**Algorithm 1.** The Optimizing Procedure of the TCNN-MGCHN.

**Input:** A labeled IMU-based skeleton graph data set $\left\{X,Y\right\}={\left\{x_{i},y_{i}\right\}}_{i=1}^{N}$, epoch parameter $E_{p}$, batch size parameter $B_{a}$, and model hyper-parameters Θ.

**Output:** The optimal parameter Θ and the learned adjacency matrix *A*.

1: **for** *e* = 1: *Ep* **do**

2:   **for** *b* = 1: *Ba* **do**

3:   Sample input $x_{e,b}$ and $y_{e,b}$ from {X,Y}.

4:   Calculate time domain feature $F_{TCM}^{\left(2\right)}$ by feeding $x_{e,b}$ into the TCM according to Eq. (1).

5:   Calculate the Laplacian matrix *L* according to Eq. (3).

6:   Calculate the spatial features $S^{*}$ by $x_{e,b}$ feeding into MGCM according to Eqs. (7) (8).

7:   Obtain label ${\hat{y}}_{e,b}$ by passing $FST^{*}$ into classification module according to Eqs. (9) (10).

8:   Use $y_{e,b}$ and ${\hat{y}}_{e,b}$ to calculate the loss function according to Eq. (11).

9:   Update the adjacency matrix *A* and model parameters Θ via adam optimizer based on the loss function.

10:   **end for**

11: **end for**

**Classification module:** Firstly, we concatenate the features $F_{MGCM}$ and $F_{TCB}^{\left(2\right)}$ to obtain the fused spatio-temporal feature *FST* . *FST*  is further refined between channel dimensions through a convolutional layer (both with a convolution kernel size of 1×1) to obtain the final spatio-temporal features *F* :

$$F^{*}=Conv\left(FST^{*}\right)\in R^{N\;\times \;S\;\times \;5T/4\;\times \;W},$$ (9)

where Conv(·) is the convolution operation. Secondly, the feature map *F*  is sequentially passed through a FC layer and a Softmax layer to obtain predicted labels $\hat{y}$:

$$\hat{y}=Soft\max\left(W_{y2}\left(ReLU\left(W_{y1}F^{*}+b_{y1}\right)\right)+b_{y2}\right),$$ (10)

where $W_{y1}$, $b_{y1}$, $W_{y2}$ and $b_{y2}$ are learned parameters, and $\hat{y}\in R^{N}$ is the predicted label of the model.

### Implementation details for TCNN-MGCHN model

Consider a IMU-based skeleton graph data set $\left\{X,Y\right\}={\left\{x_{i},y_{i}\right\}}_{i=1}^{N}$ is given, where $X\in R^{N\;\times \;S\;\times \;C\;\times \;T}$ and $Y\in R^{N}$. Initially, $x_{i}$ is transformed to $F_{TCM}^{\left(2\right)}$ using the TCM, as described in Eq (1). Next, $x_{i}$ is further transformed into $F_{MGCM}$ using the MGCM based on Eqs (7) and (8). Then, the fused spatial-temporal feature representations *FST*  by feeding $F_{TCM}^{\left(2\right)}$ and $F_{MGCM}$ into the classification module, and the prediction label $\hat{y}$ is calculated by passing *FST*  into the classification module according to Eqs (9) and (10). The detailed steps of the model optimization process are provided in Algorithm 1.

Here, cross-entropy loss *L* was used to evaluate the discrepancy between the true label $y_{i}$ and the predicted label $\hat{y}$. The calculation formula is provided below.

$$L=-\frac{1}{M}\sum_{k=1}^{K}\sum_{i=1}^{M}y_{i}^{k}\log\left({\hat{y}}_{i}^{k}\right)+\alpha {\left\|𝛩\right\|}_{1}+\beta {\left\|A\right\|}_{1},$$ (11)

where *α* and *β* are constants, Θ denotes the hyper-parameters, and ${\left\|\cdot \right\|}_{1}$ denotes the *l*_1_ norm. In Eq (11), *α* and *β* assigned values of 0.001 and 0.2, respectively, to facilitate dynamic updating of adjacency matrix *A* and mitigate overfitting. The Adam optimizer is employed with a learning rate of 0.001, and the batch size is set to 64. The TCNN-MGCHN model is implemented using Python 3.6 and PyTorch 1.9, and trained on an NVIDIA RTX 2080 Ti GPU.

## Results

We conduct an in-depth experimental analysis to demonstrate the advantages of the proposed model. The evaluation focuses on three key aspects. First, we assess the impact of model parameters and different partitioning strategies on performance. Second, detailed ablation experiments are carried out to validate the contribution of each component, specifically the TCM and MGCM, within the TCNN-MGCHN model. Lastly, we compare the proposed model against mainstream approaches to highlight its effectiveness.

To ensure accurate evaluation, we adopt the 5-fold cross-validation method. Additionally, the model’s performance in classifying gait and sub-phases is measured using four standard metrics: precision, recall, accuracy, and Matthews correlation coefficient (MCC) [41]:

$$Accuracy=\frac{TP+TN}{TP+TN+FP+FN}.$$ (12)

$$Recall=\frac{TP}{TP+FN}.$$ (13)

$$Precision=\frac{TP}{TP+FP}.$$ (14)

$$MCC=\frac{TP\;\times \;TN-FP\;\times \;FN}{\sqrt{\left(TP+FP\right)\left(TP+FN\right)\left(TN+FP\right)\left(TN+FN\right)}}.$$ (15)

where *TP*, *TN*, *FP*, and *FN* denote true positive, true negative, false positive, and false negative, respectively.

### Impact analysis of model parameters and partitioning strategies

It is well known that model parameters and partitioning strategies can significantly impact on classification performance. The convolution kernel parameter determines the receptive field of the model, affecting both the scope of temporal information analyzed from the angular velocity signal and the computational complexity. Therefore, it is important to explore how to balance the information extraction capability of the TCNN-MGCHN model with computational efficiency effectively. Table 1 presents the model’s performance using various multi-scale convolution kernel parameters for the TCM, including [1×1, 1×3, 1×5], [1×1, 1×3, 1×7], [1×1, 1×5, 1×7], and [1×3, 1×5, 1×7]. The results indicate that the TCNN-MGCHN model achieves optimal classification performance for both gait and gait sub-phase tasks when the multi-scale convolution kernel parameters are set to [1×1, 1×5, 1×7]. In particular, the accuracy, recall, precision, MCC for the parameters [1×1, 1×5, 1×7] on the gait classification task are 98.24%±0.24, 98.53%±0.30, 98.12%±0.26, and 98.13%±0.20, respectively. The average accuracy, recall, precision, MCC for parameter [1×1, 1×5, 1×7] are 97.09%±0.65, 97.43%±0.82, 96.96%±0.47 and 97.15%±0.58, respectively.

**Table 1:** Comparison of different convolution kernel parameters.

Classification task	[1×1, 1×3, 1×5]			
	Accuracy(%)	Recall(%)	Precision(%)	MCC(%)
Gait	96.89±0.44	96.36±1.40	96.28±1.73	96.22±1.05
Normal walking (sub-phase)	95.47±1.12	95.78±2.65	94.45±2.69	95.46±2.15
Downstairs (sub-phase)	**97.21±1.45**	**97.58±2.02**	94.56±2.45	94.26±3.69
Upstairs (sub-phase)	95.28±0.89	96.24±4.02	95.89±2.48	**96.29±2.16**
Running (sub-phase)	95.12±1.56	94.47±2.56	**96.45±3.02**	95.78±2.23
**Average**	95.74±1.25	95.51±3.05	95.62±2.58	95.14±2.72
Classification task	[1×1, 1×3, 1×7]			
	Accuracy(%)	Recall(%)	Precision(%)	MCC(%)
Gait	**96.47±0.41**	96.36±0.66	96.47±0.78	95.74±0.62
Normal walking (sub-phase)	95.32±1.42	95.32±2.45	94.56±1.48	95.58±2.67
Downstairs (sub-phase)	96.29±1.85	**97.26±2.51**	94.26±3.45	93.26±1.59
Upstairs (sub-phase)	96.32±0.49	96.36±2.21	95.58±1.48	**96.47±1.72**
Running (sub-phase)	94.22±1.16	94.45±1.23	**96.79±3.23**	95.26±1.23
**Average**	95.30±1.20	95.20±1.51	95.04±2.34	94.62±2.26
Classification task	[1×1, 1×5, 1×7]			
	Accuracy(%)	Recall(%)	Precision(%)	MCC(%)
Gait	**98.24±0.24**	**98.53±0.30**	**98.12±0.26**	**98.13±0.20**
Normal walking (sub-phase)	97.47±1.21	96.78±1.34	96.75±1.19	96.56±1.05
Downstairs (sub-phase)	97.25±1.92	97.43±1.56	95.78±1.15	97.26±1.39
Upstairs (sub-phase)	96.58±0.45	96.78±1.32	95.86±0.78	96.75±1.21
Running (sub-phase)	96.49±1.45	96.97±1.56	97.45±1.02	96.78±1.03
**Average**	97.09±0.65	97.43±0.82	96.96±0.47	97.15±0.58
Classification task	[1×3, 1×5, 1×7]			
	Accuracy(%)	Recall(%)	Precision(%)	MCC(%)
Gait	**96.85±0.56**	96.72±1.53	**96.75±1.98**	**96.63±1.29**
Normal walking (sub-phase)	95.27±1.05	95.84±2.78	95.45±1.58	95.59±2.15
Downstairs (sub-phase)	96.25±1.42	**96.94±2.45**	94.89±3.55	95.42±3.69
Upstairs (sub-phase)	94.23±0.79	96.34±2.23	95.47±3.59	96.13±2.16
Running (sub-phase)	95.23±1.58	94.78±1.56	96.65±2.42	95.82±2.23
Average	95.70±1.48	95.55±3.47	95.63±3.05	95.66±2.92

*Note*: Bold font indicates the maximum value for each column.

As mentioned above, we designed three partitioning strategies to facilitate the model to effectively mine the internal spatial dependence information between skeleton nodes in different body regions. Therefore, we explore the impact of three partitioning strategies on model performance, as shown in Table 2. Experimental results indicate that the TCNN-MGCHN model achieves the lowest average recognition accuracy, 88.68%±1.42, when using the uni-label partitioning strategy. In addition, the dual-label partitioning strategy outperformed the uni-label partitioning strategy in all evaluation metrics. The body partitioning strategy helps the most in improving the model performance. The average accuracy, recall, precision, and MCC of the gait and sub-phase classification tasks are 97.09%, 97.43%, 96.96%, and 97.15%, respectively. Moreover, to validate the statistical significance of the performance differences among the three partitioning strategies, we conducted paired *t*-tests on accuracy, rcall, precision, and MCC across five sub-tasks. The results revealed that the body partitioning strategy significantly outperformed both the dual-label (*p*<0.05) and uni-label (*p*<0.01) strategies across all metrics. The dual-label strategy also consistently outperformed the uni-label strategy (*p*<0.05). These findings confirm that explicitly modeling region-specific dependencies among skeletal joints contributes to better recognition performance.

**Table 2:** Comparison of different partitioning strategies.

Classification task	Uni-label partitioning strategy			
	Accuracy(%)	Recall(%)	Precision(%)	MCC(%)
Gait	**91.32±0.56**	**92.49±2.27**	88.42±1.75	87.52±0.89
Normal walking (sub-phase)	86.42±1.59	91.32±2.03	87.26±1.63	**88.49±1.56**
Downstairs (sub-phase)	87.45±1.49	85.94±1.26	87.29±2.15	85.16±1.69
Upstairs (sub-phase)	90.43±0.82	90.25±1.23	**89.36±1.52**	86.01±1.04
Running (sub-phase)	90.13±1.89	89.67±2.89	88.26±1.59	85.47±1.22
Average	88.68±1.42	89.73±2.43	88.63±1.58	86.31±1.22
Classification task	Dual-label partitioning strategy			
	Accuracy(%)	Recall(%)	Precision(%)	MCC(%)
Gait	**96.54±0.53**	**97.42±0.79**	**95.73±0.78**	**94.42±0.62**
Normal walking (sub-phase)	95.11±1.24	95.46±1.49	93.72±1.16	92.14±2.57
Downstairs (sub-phase)	94.29±1.16	95.24±1.69	92.49±2.44	91.46±2.26
Upstairs (sub-phase)	93.59±0.99	96.59±2.16	94.47±2.16	92.47±2.04
Running (sub-phase)	94.46±1.25	94.16±1.69	93.18±1.26	91.67±2.18
Average	94.32±1.11	95.66±1.63	93.44±1.39	92.49±2.26
Classification task	Body partitioning strategy			
	Accuracy(%)	Recall(%)	Precision(%)	MCC(%)
Gait	**98.24±0.24** ^**^	**98.53±0.30** ^**^	**98.12±0.26** ^**^	**98.13±0.20** ^**^
Normal walking (sub-phase)	96.48±1.14^*^	96.99±0.96^*^	95.04±0.57^*^	96.72±0.72^*^
Downstairs (sub-phase)	95.89±1.12^*^	96.69±1.15^*^	95.36±0.56^*^	96.69±0.64^*^
Upstairs (sub-phase)	96.79±0.88^**^	97.69±1.33^**^	96.72±0.69^**^	96.59±0.69^**^
Running (sub-phase)	96.85±1.18^**^	98.36±1.14^**^	97.63±0.41^**^	97.94±0.36^**^
Average	97.09±0.65^**^	97.43±0.82^**^	96.96±0.47^**^	97.15±0.58^**^

*Note*: Bold font indicates the highest value for each column. ^*^ and ^**^ indicate statistically significant improvement over the dual-label and uni-label strategies, respectively (*p* < 0.05 and *p* < 0.01), based on paired *t*-tests across five sub-tasks.

### Impact analysis of multi-branch graph convolutional network architecture

**Fig 8:**
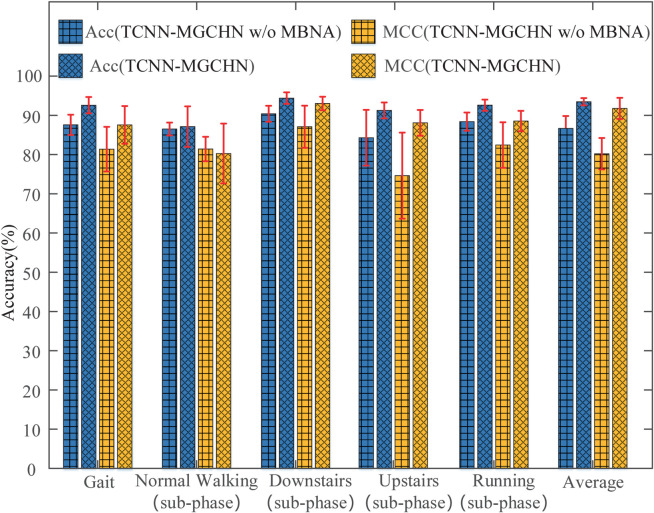
Accuracy and MCC of TCNN-MGCHN and TCNN-MGCHN w/o MBNA models. The red line represents the standard deviation.

To assess the impact of the multi-branch network structure in the proposed MGCM, we compare the performance of two models, TCNN-MGCHN and TCNN-MGCHN w/o multi-branch network architecture (TCNN-MGCHN w/o MBNA). The TCNN-MGCHN w/o MBNA model only retains the backbone structure and removes two branch structures. As illustrated in Fig 8, the TCNN-MGCHN model achieves an average accuracy that is 5.44% higher compared to the TCNN-MGCHN w/o MBNA model. Moreover, a notable difference in accuracy between the two models is observed in the upstairs sub-phase classification task, where the TCNN-MGCHN model outperforms the TCNN-MGCHN w/o MBNA model by 10.6%. In terms of MCC performance indicators, the TCNN-MGCHN model is also basically better than the TCNN-MGCHN w/o MBNA model except for the normal walking sub-phase classification task. The average MCC of the TCNN-MGCHN model is 8.50% higher than that of the TCNN-MGCHN w/o MBNA model. These findings demonstrate that the multi-branch structure effectively enhances the recognition performance of the TCNN-MGCHN model in both gait classification and gait sub-phase classification tasks.

**Fig 9:**
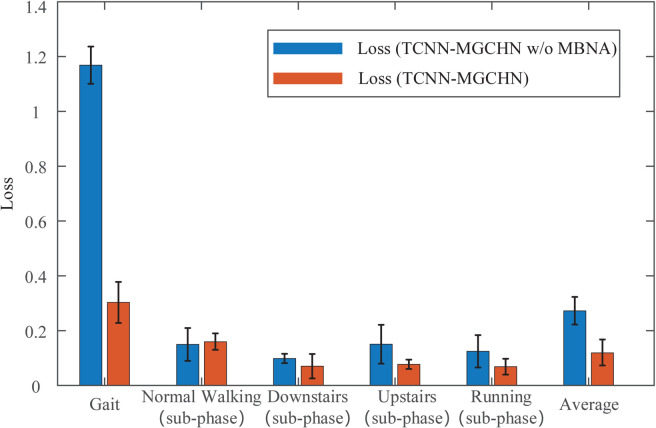
Loss value of TCNN-MGCHN and TCNN-MGCHN w/o MBNA models. The black line represents the standard deviation.

In addition, we calculate the loss values of the TCNN-MGCHN and TCNN-MGCHN w/o MBNA models under the seven classification tasks, as shown in Fig 9. The loss value provides a more reliable measure of the error between the classifier’s output and the true label compared to the accuracy metric. As shown in Fig 9, the TCNN-MGCHN model consistently exhibits a lower loss value than the TCNN-MGCHN w/o MBNA model, except in the normal walking sub-phase classification task. Especially for the gait classification task, the loss value of the TCNN-MGCHN model is 0.8656 lower than that of the TCNN-MGCHN w/o MBNA model.

### Ablation study

**Fig 10:**
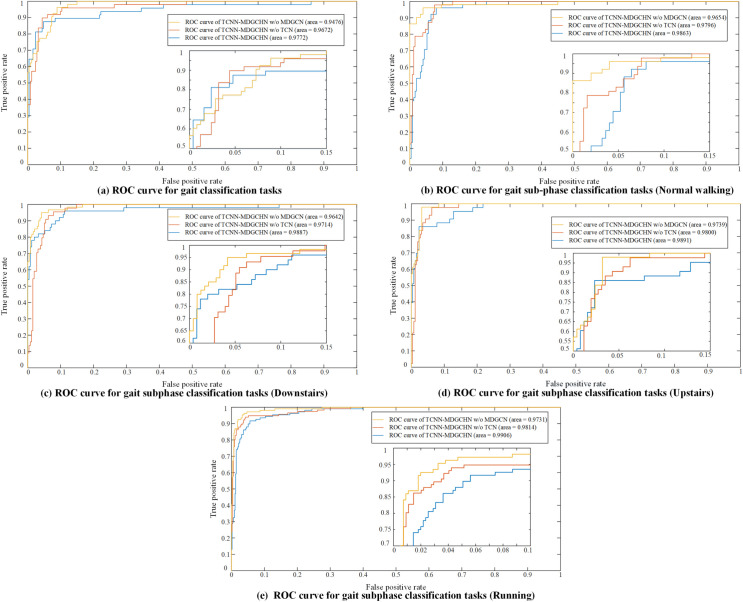
Comparison of AUC curves for ablation studies.

Here, the importance of each module of the TCNN-MGCHN model is validated by ablation experiments: the TCM and MGCM. As shown in Fig 10, it can be found that the average area under curve (AUC) values of the TCNN-MGCHN model are all improved compared to TCNN-MGCHN w/o TCM and TCNN-MGCHN w/o MGCM.

The above results show that both TCM and MGCM contribute significantly to the classification performance improvement of the TCNN-MGCHN model, which is closely related to TCM capturing temporal information and MGCM excelling in mining spatial dependence information among various skeleton nodes. Consequently, the classification performance of the TCNN-MGCHN model is negatively affected if any of the functional modules are discarded. Furthermore, MGCM is the most helpful in improving the model performance among the two modules, with AUC increased by 2.96% (gait), 2.09% (normal walking sub-phase), 2.45% (downstairs sub-phase), 1.52% (upstairs sub-phase), and 1.75% (running sub-phase), respectively. Compared with the TCNN-MGCHN w/o TCN model, the AUC of the TCNN-MGCHN model is increased by 1% (gait), 0.67% (normal walking sub-phase), 1.73% (downstairs sub-phase), 0.91% (upstairs sub-phase), and 0.92% (running sub-phase), respectively.

**Fig 11:**
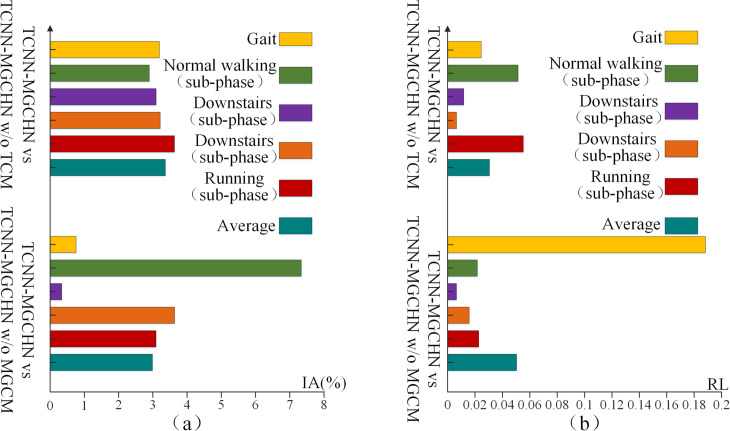
Performance comparison of TCNN-MGCHN with TCNN-MGCHN w/o TCM and TCNN-MGCHN w/o MGCM. (a) The improved accuracy (IA). (b) The reduced loss (RL) value.

Fig 11 illustrates the increased accuracy (IA) and reduced loss (RL) of the TCNN-MGCHN model compared to TCNN-MGCHN w/o TCM and TCNN-MGCHN w/o MGCM across all classification tasks. In Fig 11(a), the improvement in accuracy of the TCNN-MGCHN model relative to the TCNN-MGCHN w/o MGCM model varies depending on the classification task. The largest improvement of 7.34% is achieved on the normal walking sub-phase classification task, and the smallest improvement of 0.34% is achieved on the downstairs sub-phase classification task. In addition, the improved accuracy of the TCNN-MGCHN model relative to the TCNN-MGCHN w/o TCM model is close on different classification tasks, with an average improvement of 3.17%. As shown in Fig 11(b), the TCNN-MGCHN model has the largest reduced loss value of 0.19 relative to the TCNN-MGCHN w/o MGCM in the gait classification task. The reduced loss value of the TCNN-MGCHN model relative to the TCNN-MGCHN w/o MGCM model is not greater than 0.03 on other sub-phase classification tasks. In addition, on the normal walking and downstairs classification tasks, the TCNN-MGCHN model has the largest reduced loss values relative to the TCNN-MGCHN w/o TCM model, which are 0.55 and 0.51, respectively. Similarly, the reduced loss value of the TCNN-MGCHN model relative to the TCNN-MGCHN w/o MGCM model is no more than 0.03 on other classification tasks. These findings suggest that both TCM and MGCM independently enhance the model’s recognition capabilities, with their combination leading to further improvements in accuracy and robustness. The combination of these components proves critical for optimizing both the performance and efficiency of the TCNN-MGCHN model across a wide range of gait recognition tasks.

### IMU contribution visualization for gait recognition

**Fig 12:**
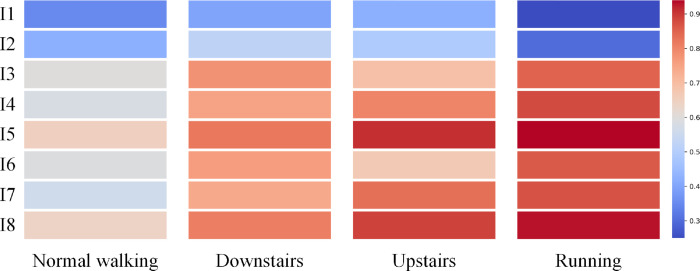
Contribution heatmap of individual IMU sensors across different gait classes.

To further enhance the interpretability of the proposed model, we visualize the contribution of each IMU sensor to the classification of different gait types (i.e., normal walking, upstairs, downstairs, and running) in the form of a contribution heatmap, as shown in Fig 12. The matrix presents the average importance scores of the eight IMU sensors, which are computed using a gradient-based attribution method across all correctly classified samples. It can be observed that the sensors placed on the lower limbs—particularly those on the feet (I5/I8) and shanks (I4/I7)—exhibit higher contribution scores across all gait categories. This is especially evident in dynamic movements such as running and stair descent, where the foot-ground interaction and leg motion are more pronounced. In contrast, the sensors on the upper body (I1 on the chest and I2 on the abdomen) contribute relatively less, but still provide valuable information for posture and balance, particularly in walking and downstairs. The visualization confirms the rationality and effectiveness of the proposed architecture in leveraging spatial information from multi-IMU input for accurate gait recognition.

### Comparison with some mainstream classification algorithms

Like the proposed TCNN-MGCHN model, other studies have also achieved impressive results in the field of gait recognition. Table 3 presents a detailed performance comparison between the TCNN-MGCHN algorithm and several widely adopted methods in this domain. To ensure a fair comparison, we applied each of these methods to the IMU-based skeleton graph dataset collected in this study. The methods included in the comparison are DBN [42], Phase Variable [43], Bi-LSTM [44], CNN [45], ConvLSTM [46], DAFO [28], LDA-PSO-LSTM [27], as well as the recently proposed DPF-LSTM-CNN [47], LSTM-CRF [48], LSTM-CRF [49], and Bi-LSTM [50]. By applying these algorithms to the same IMU-based skeleton graph dataset, we ensured consistency and reliability in the performance assessment. Additionally, we employed uniform preprocessing across all models, and the evaluation was based on the same performance metric: accuracy. This approach provides an unbiased and comprehensive comparison of the methods.

**Table 3:** Comparison with some state-of-the-art methods.

Author	Year	Method	Accuracy(%)
Hassan et al. [42]	2018	DBN	85.61
Bartlett et al. [43]	2018	Phase Variable	87.32
Turner et al. [44]	2019	Bi-LSTM	91.80
Rohan et al. [45]	2020	CNN	92.31
Lu et al. [46]	2021	ConvLSTM	92.22
Zhang et al. [28]	2022	DAFO	95.58
Cai et al. [27]	2022	LDA-PSO-LSTM	94.17
Liu et al. [47]	2023	DPF-LSTM-CNN	94.75
Wei et al. [48]	2023	LSTM-CRF	95.02
Jung et al. [49]	2024	LSTM-RNN	92.84
Jeon et al. [50]	2024	Bi-LSTM	85.79
**This paper**	**-**	**TCNN-MGCHN**	**97.54**

As shown in Table 3, the TCNN-MGCHN model achieves the highest accuracy on the custom dataset, reaching an impressive 97.54%. This result is notably 11.93%, 10.22%, 5.74%, 5.23%, 5.32%, 1.96%, and 3.37% higher than the accuracy rates achieved by the DBN, Phase Variable, Bi-LSTM, CNN, ConvLSTM, DAFO, and LDA-PSO-LSTM models, respectively. This highlights the superior performance of the TCNN-MGCHN model in the context of IMU-based gait recognition, making it a significant advancement in this area. In conclusion, while the mainstream methods compared in this study have demonstrated solid performance in gait recognition, our work introduces a valuable contribution by providing a complementary approach that offers improved results. Specifically, 1) The TCNN-MGCHN model demonstrates the highest recognition accuracy, positioning it as an ideal method for driving future research and deep-learning modeling efforts related to IMU-based gait and sub-phase recognition. 2) The body partitioning strategy proposed in this study enhances the spatial feature extraction capability of the MGCM, which not only improves recognition accuracy but also holds potential for broader applications. Furthermore, this body partitioning strategy can be extended to other IMU-based GCN modeling works, enabling a more comprehensive understanding and analysis of human motion. Thus, this work sets a new benchmark in the field and paves the way for further research and innovation in the area of IMU-based gait recognition.

### Comparison of model parameters

The average inference time per sample is approximately 13.25 ms, and the total parameter size of the proposed model is about 23.72 MB. While this is not the smallest among existing methods, it represents a relatively compact architecture with competitive recognition performance. As shown in Table 4, models specifically designed for lightweight deployment, such as SkeletonGait[36] (10.87 MB), NSVGT-ICBAM-FACN[37] (13.60 MB), and GPGait[38] (9.66 MB), typically have smaller parameter sizes below 15 MB. In contrast, non-lightweight models generally require much larger storage, such as GaitRGA[39] with 76.37 MB and GaitGL[40] with 30.72 MB. The proposed model, with its 23.72 MB parameter size, is larger than the highly lightweight designs but remains considerably smaller than heavy-weight architectures like GaitRGA and GaitGL, thereby achieving a balance between model compactness and recognition capability, while achieving superior recognition performance (see Table 3 for accuracy comparisons).

**Table 4:** Comparison of model parameters of different gait recognition models.

Author	Year	Method	Parameters(MB)
Simonyan et al. [40]	2022	GaitGL	30.72
Fu et al. [38]	2023	GPGait	9.66
Liu et al. [39]	2024	GaitRGA	76.37
Li et al. [37]	2024	NSVGT-ICBAM-FACN	13.60
Fan et al. [36]	2025	SkeletonGait	10.87
This paper	-	TCNN-MGCHN	23.72

### Limitation

It is important to note that all participants in this study were healthy individuals. In real-world applications, gait patterns may be significantly altered in pathological populations, such as individuals with neurological diseases (e.g., Parkinson’s disease or stroke). These altered movement characteristics may challenge the model’s generalization ability, especially if it is trained solely on healthy data. To enhance robustness and applicability, future work should include subjects with gait impairments and consider incorporating domain adaptation or transfer learning techniques to improve performance in clinical scenarios. Moreover, although this study focuses solely on angular rate signals as the analytical physical quantity, we recognize that integrating linear acceleration may further improve recognition performance, particularly for non-cyclic or complex motion patterns. However, given the cyclic nature of gait and sub-phase movements, angular rate alone provided sufficient discriminatory power in our tasks. Future work will explore the fusion of angular and linear kinematic features to enhance model robustness and generalizability across a wider range of human activities.

## Conclusion

This study designed a multi-task gait experiment and constructed IMU-based skeleton graph data. We also propose a multi-branch deep learning network, TCNN-MGCHN, for accurate gait and sub-phase recognition using this data. The model comprises two main components: TCM and MGCM. Initially, temporal feature representations of angular velocity signals are extracted through the TCM. Subsequently, the MGCM captures intrinsic spatial dependencies between skeleton nodes. Finally, the fused temporal and spatial features are fed into the classification module for gait and sub-phase prediction. The TCNN-MGCHN model’s classification accuracy and robustness are comprehensively evaluated using mathematical statistics and performance assessment, including overall performance, parameter analysis, and ablation studies. With an accuracy of 97.54%, the TCNN-MGCHN model outperforms seven mainstream methods. These results demonstrate the superior recognition performance and generalization capability of the proposed model. Also, the body partitioning strategy proposed in this paper can focus on crucial channels and skeleton nodes, which is conducive to enhancing the ability of MGCM to mine discriminating information. Despite the robustness and superiority of our proposed model, some problems still need to be improved. Firstly, our proposed IMU-based skeleton graph data construction method is relatively simple, which cannot learn the interaction information between skeleton nodes from multiple perspectives. In future work, we will explore more sophisticated and rational methods of constructing IMU-based skeleton graph data. Secondly, we only consider four gait and sub-phases. More types of gait and sub-phases identification methods will be studied.

## Declaration

The study was conducted in accordance with the Declaration of Helsinki, and the experimental protocol was approved by the Human Ethical Review Committee of Zhejiang University of technology (Approval No. 2023-314).
